# The case of the disappearing librarians: analyzing documentation of librarians' contributions to systematic reviews

**DOI:** 10.5195/jmla.2022.1505

**Published:** 2022-10-01

**Authors:** Amelia Brunskill, Rosie Hanneke

**Affiliations:** 1 abrunsk2@uic.edu, Assistant Professor and Information Services & Liaison Librarian, University of Illinois Chicago, Chicago, IL; 2 rhanneke@uic.edu, Associate Professor & Head, Information Services & Research, University of Illinois Chicago, Chicago, IL

**Keywords:** Systematic reviews, librarians, documentation

## Abstract

**Objective:**

The study aimed to analyze the documented role of a librarian in published systematic reviews and meta-analyses whose registered protocols mentioned librarian involvement. The intention was to identify how, or if, librarians' involvement was formally documented, how their contributions were described, and if there were any potential connections between this documentation and basic metrics of search reproducibility and quality.

**Methods:**

Reviews whose PROSPERO protocols were registered in 2017 and 2018 and that also specifically mentioned a librarian were analyzed for documentation of the librarian's involvement. Language describing the librarian and their involvement was gathered and coded, and additional information about the review, including search strategy details, was also collected.

**Results:**

A total of 209 reviews were found and analyzed. Of these, 28% had a librarian co-author, 41% named a librarian in the acknowledgements section, and 78% mentioned the contribution of a librarian within the body of the review. However, mentions of a librarian within the review were often generic (“a librarian”) and in 31% of all reviews analyzed no librarian was specified by name. In 9% of the reviews, there was no reference to a librarian found at all. Language about librarians' contributions usually only referenced their work with search strategy development. Reviews with librarian coauthors typically described the librarian's work in active voice centering the librarian, unlike reviews without librarian coauthors. Most reviews had reproducible search strategies that utilized subject headings and keywords, but some had flawed or missing strategies.

**Conclusion:**

Even among this set of reviews, where librarian involvement was specified at the protocol level, librarians' contributions were often described with minimal, or even no, language in the final published review. Much room for improvement appears to remain in terms of how librarians' work is documented.

## INTRODUCTION

Participation in a systematic review can represent a considerable time investment for librarians [1] and there is a multitude of roles that a librarian can play within a systematic review [2]. Yet while official documentation of this effort can be important in performance evaluations [3], and librarian co-authorship has been found to correlate with higher quality reported search strategies [4–6], librarians' contributions sometimes fade into the background as invisible labor, described in the final publication without specific named recognition of the librarian who performed the work [7].

Multiple studies have explored the documentation of librarians' work in systematic reviews. Their approaches, however, have differed, with some studies surveying authors of systematic reviews about librarians' role in the work [5,8,9], some surveying librarians themselves about their experiences [10], and others focusing on analyzing the text of systematic reviews for evidence of the librarian's role [4,6,11,12]. Reviews of librarian documentation within a publication have usually involved checking for a librarian in three places: among the authors, in the body of the text, and in the acknowledgements [4, 6, 12]. Mention of a librarian in the acknowledgements or in the body of a paper usually occurred more frequently than as authors, and no mention of a librarian at all was often the most common scenario [4, 6, 12]. The prevalence of librarian co-authorship varied considerably by study, with a high of almost 20 percent found in a study of the impact of a library's tiered systematic review service [12] to a low of just over one percent in a study of work affiliated with another institution [4].

The scope for previous studies on librarian documentation in systematic reviews has often been intentionally limited by elements such as journal impact factor, subject matter, or author affiliation with a specific institution. The aim of this study was to take an approach that avoided such limitations, and instead identified recent reviews where librarian involvement had been specifically indicated by the authors prior to the publication of the review. We thought that PROSPERO, an international database where researchers can prospectively register their systematic review and the details of their intended search strategy for topics pertaining to health and social care, or other areas with a health-related outcome [13], would be an excellent match for this purpose. As a repository of information about systematic reviews, PROSPERO has some particular advantages for studying librarian documentation: registration in PROSPERO is already considered a best practice [14], authors are supposed to update their PROSPERO records with citation information when a review is published, and, while there is no formal enforcement, these protocols are intended to serve as a specific commitment to the described approach, so statements about librarian involvement should represent actual involvement for the review.

No articles were found within the library literature that used data from PROSPERO either as a data set or to identify a set of records for the study. However, other disciplines have done so, analyzing PROSPERO data for information such as planned use of risk of bias tools [15], study eligibility criteria [16], and adherence to reporting guidelines [17].

The research questions of interest for this study were as follows:

- How were librarian contributions documented in the final published review?
- What language was used to describe the librarians and the work that they contributed?
- Was there a connection between the reproducibility and quality of the search strategy and the level of documentation of the librarian?

Together, these questions will provide additional insight into current practices in documenting and discussing librarians' contributions to systematic reviews, and what advocacy may still be needed in this area.

## METHODS

### Identification of reviews

**Figure 1:**
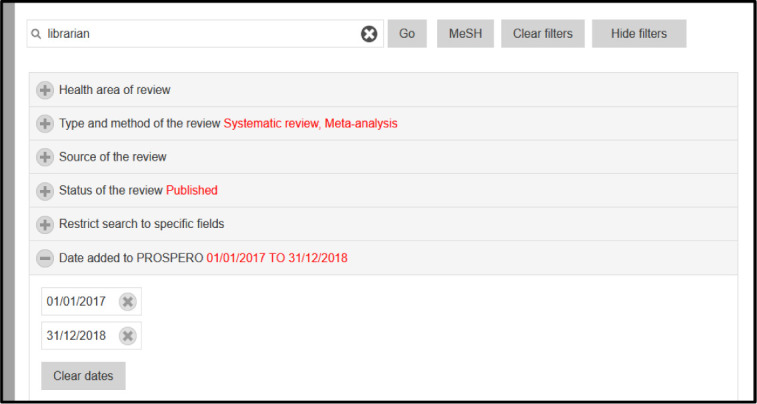
Screenshot of search approach in PROSPERO.

On August 2, 2021, we searched PROSPERO for protocols that matched the following criteria: they mentioned a librarian anywhere in the text of their protocol; were listed as either a systematic review or meta-analysis; had an updated status of published; and were registered in 2017 or 2018 (see Figure 1). The dates of 2017 and 2018 were selected to focus on recently initiated reviews, which had still had over two years after registration for the authors to complete and publish the review.

While the initial hope had been to use PROSPERO's export option, that option only included the date that the review was registered, the authors of the review, and the title of the review. No information about the registration number for the review, nor information about the final publication for the review was included. Since the registration number for the review was included as part of the title in the list of results returned on the PROSPERO search, the list of all returned protocols was copied and pasted into Excel. Then the registration numbers were extracted from the title field and used to generate links back to the individual protocol records, and links to the final publications were manually extracted from each protocol record.

PROSPERO records were excluded if no publication was listed in the PROSPERO record, if a faulty link was provided, or if the final publication was not structured in the format of a traditional journal article, such as a government or agency report.

We located the published reviews associated with the PROSPERO protocols using the manually extracted link to the review.

### Data extraction

We created a Qualtrics form for extracting information from each review including information about the documentation of library authorship, any language about a librarian in the body of the review or in the acknowledgements, and information provided about the search strategy. After piloting the form together to help ensure a consistent extraction approach, we split the full set of reviews in half and we each extracted the data for half of the reviews.

Librarian authorship was initially checked by reviewing the authors' degrees and affiliations listed in the published reviews. For reviews in which no librarian was identified within the list of authors, or listed in the acknowledgements, online searches were conducted for all authors unless their listed degrees and affiliations were clearly associated with another profession, such as an MD. For documentation within the body of the review, a search was conducted for “librarian” and the methods section was also specifically reviewed in case another term, such as “information specialist” was used. All references to a librarian were copied from the text of the review into the Qualtrics form. The acknowledgements section was searched in each review, and any references to a librarian were again copied into the form.

Descriptions of the search strategy were reviewed for information about the number of databases searched as well as details of the search strategy, including a reproducible search strategy for a named database that included Boolean operators, and the inclusion of subject headings and keywords. As in Rethlefsen's study [6], searches were checked for at least one complete search strategy that included Boolean logic. If this was found, we would consider the search reproducible if information about the database was also included.

The descriptions of librarians and of their contributions were compiled and reviewed. We then reviewed these descriptions for individual words used to describe the librarian's position and/or expertise and compiled these for analysis. Descriptions of librarians' contributions were coded using the codes in Table 1. Codes were developed using the approach proposed by Ross-White as an initial starting point. Ross-White posited that search used as an active verb/noun gave agency to the searcher while the use of the passive voice minimized the searcher's role [7]. We expanded our coding to identify appropriate codes for language that did not cleanly match the previously established codes, and also to account for some differences in how work was described in the body of the manuscript versus in the acknowledgments. We coded an initial set together, and subsequent coding was split between us to complete individually. We then compiled a list of individual words used to describe the librarian's position and/or expertise and counted each term's frequency across the reviews.

**Table 1:** Codes for language about librarian's contributions.

Code	Used to code language in…	Used for language that…	Example(s)
librarian active voice	manuscript & acknowledgements	indicated that the librarian had primary ownership over the work in question	“A librarian designed and executed the searches” “Many thanks to [name of librarian] for constructing and implementing the search.”
author team active voice	manuscript	the authors had primary ownership over the work, and were assisted by a librarian	“We designed the searches after consulting with a librarian”
Collaborative	manuscript	indicated the librarian had a partnership role in the activity, such as working alongside the authors or taking on a role of authority among those working on a task (“led”, “supervised”) but not executing it themselves.	“The librarian and the first author constructed the search strategies.” “The design and execution of the searches were supervised by a research librarian”
helper or consultant role	acknowledgements	librarian was acknowledged for assisting or consulting with the author team	“We thank [name of librarian] for providing feedback on our search strategy”
passive voice	manuscript	used passive voice to describe the work	“The published literature was searched”
mixed	manuscript	librarian's work is described in more than one voice, such as passive for search strategy and active for conducting the search	“A search strategy was developed and then a librarian implemented the search.”
*N/A*	Acknowledgements	a librarian was named in acknowledgments without any description of their tasks or role	“Many thanks also to [name of librarian] for their assistance.”

## RESULTS

### Basic descriptive information about the reviews

Initially, 213 protocols met our search criteria. Among these, ten were excluded, four because the publication listed was not a journal article, three because they did not list publication information, two because they did not provide a working link to the review, and one because it listed a review published prior to the registered protocol. Of the remaining 203 protocols, six of them resulted in two separately published reviews, so ultimately, 209 systematic reviews were located and included in this study.

These reviews were published in 168 distinct journals from a wide variety of disciplines including many different areas of medicine, occupational therapy, physical therapy, nursing, mental health, dentistry, criminal justice, psychology, public health, disability studies, and information science. They were all published between 2017 and 2021.

### Documentation of librarian involvement

The three categories of documentation of librarian involvement reviewed—authorship, being named in the acknowledgements, and being mentioned in the body of the paper—were found to have common, but not completely consistent relationships. Librarians listed as co-authors were never also individually highlighted in the acknowledgements section but were often specifically mentioned in the body of the paper (47/58). Librarians listed in the acknowledgements section would usually, but not always, be referenced within the body of the paper (71/86).

**Figure 2:**
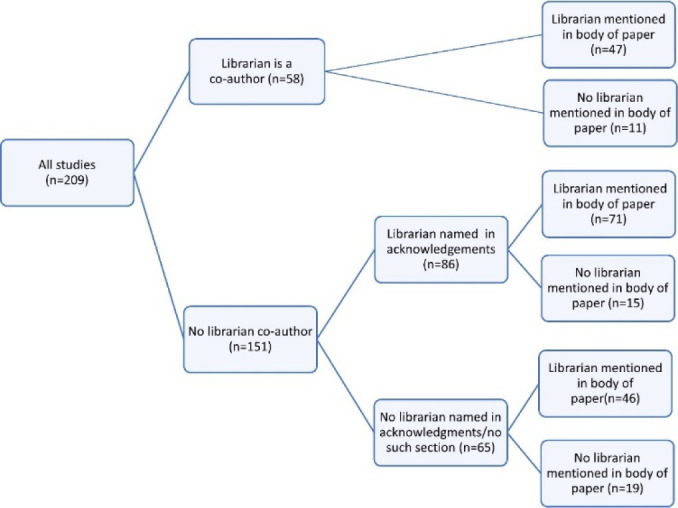
Breakdown of documentation of librarian involvement.

In five of the reviews with a librarian co-author, an additional librarian was listed in the acknowledgements section. In three of these cases, the additional librarian was specifically noted as a peer reviewer of the search strategy. These five instances were not included in the overall acknowledgment numbers, as they reflect documentation of the more limited involvement of a librarian other than the primary librarian for the study, and as such do not directly address the research question. Figure 2 shows the breakdown of documentation starting with authorship.

#### Authorship

In 58 reviews (28%), a librarian was listed as an author. There were 48 distinct librarian authors, and nine librarians were authors on multiple reviews, seven of whom were authors of reviews produced for multiple registered protocols, and two who were authors on two reviews published for the same protocol.

Librarian authors were affiliated with institutions in ten different countries (Australia, Canada, England, France, Iran, Ireland, Italy, Netherlands, Singapore, and the USA).

The largest number of reviews were from librarian authors affiliated with an institution in the United States (n=20), closely followed by librarian authors affiliated with a Canadian institution (n=19). Within the United States, librarian authors were affiliated with institutions in 12 different states.

#### Acknowledgements

In 86 reviews (41%), a librarian was mentioned in the acknowledgement section. In one additional review, support from the library was mentioned in the acknowledgements, but without the specification of an individual's name, or even the term “librarian.” As such, this was not included in the above count.

#### Body of paper

164 reviews (78%) included a mention of a librarian in the body of the review, typically within the methods section, and in 47 of these cases, a librarian was also listed as an author. In 46 reviews, there was a mention of a librarian having participated in the work, but no librarian name was included in the review as an author or in the acknowledgements.

#### No use of librarian name within the review

In 65 reviews (31%), a librarian was not mentioned by name. In 46 of these reviews, there was instead only a generic mention of a librarian (e.g., “a librarian” rather than “Rosie Hanneke”), and in the remaining 19 of these reviews, there was no reference to a librarian at all, generic or named, within the review despite having mentioned a librarian in the protocol.

### Analysis of language used about librarians and their work

#### Descriptors used for librarians

The language used to describe librarians' expertise and/or training was reviewed. Authors frequently included in their methods an allusion to the librarian's experience (e.g., “[a] literature search strategy was developed … with a librarian with 5 years' experience in conducting systematic searches” [18] or “[a]n expert health librarian…guided the search” [19]). This emerged across the reviews; however, there was little consistency in which terms were used (see Table 2).

**Table 2:** Terms used to describe a librarian's expertise.

Terms denoting experience	Frequency
experienced	24
specialist(s)	18
trained	5
expert	5
professional	5
senior	5
expertise	4
experience	3
specialised/specialized	3
independent	2
specialty	1
certified	1

#### Descriptions of librarian's work & role

Descriptions of the librarian's work in the body of the review were found in all 164 of the reviews that mentioned a librarian in the body of the review. In 162 of the reviews, the language was in the methods section, and in two reviews the librarian was only mentioned in the discussion section. In 18 reviews, the librarian was mentioned in multiple locations, including the abstract, discussion, strengths & limitations, and the author contributions section. Librarian involvement was usually cited as a strength of the study when listed in the discussion or strengths & limitations (e.g., “[o]ur search was comprehensive and supervised by an experienced research librarian” [20]). There were 86 acknowledgements that mentioned a librarian, 82 of which described that librarian's work.

As shown in Table 3, in both the body of the review and the acknowledgements, the majority discussed the work of the librarian in developing or designing search strategy, with the next largest portion (21%) using non-specific language about help with the search, and then the third largest (15%) mentioning a librarian's work with conducting or executing a search. It was rare to see mention of the librarian participating in manuscript writing, article screening or citation management, and no reviews mentioned work pertaining to deduplication, acquisition of full text, or full-text review.

**Table 3:** Description of librarian's tasks.

Description of librarian's tasks		
	In body of review (n=164)	In acknowledgements (n=86)
**Tasks described**		
Developing or designing search strategy	74% (122)	64% (55)
Search, general (design vs. execution not specified)	21% (34)	27% (23)
Conducting or executing searches	15% (24)	17% (15)
Manuscript writing or preparation	3% (5)	1% (1)
Citation management	1% (1)	2% (2)
Article screening	1% (1)	0%
**No tasks described or unclear**		
Unclear or other	1% (1)	6% (4)
Only generic thanks, no description of work	NA	6% (4)

#### Approach to language about librarian's contribution

In terms of how the work was described in the body of the review, 78 were coded as passive voice, 36 were coded as author team active voice, and 39 were coded as librarian active voice. Five studies used mixed language to describe different work by the librarian, and seven studies used collaborative voice. In the acknowledgements, the majority (86%, 74/86) used “helper or consultant role” language.

There were sometimes differences between how the work of the librarian was described in the body of the review and how their work was described in the acknowledgements. These differences included describing the librarian's work in passive voice in the body of the review and then using librarian active voice in the acknowledgements or describing additional tasks in the acknowledgements that had not been mentioned in the body of the paper.

**Figure 3:**
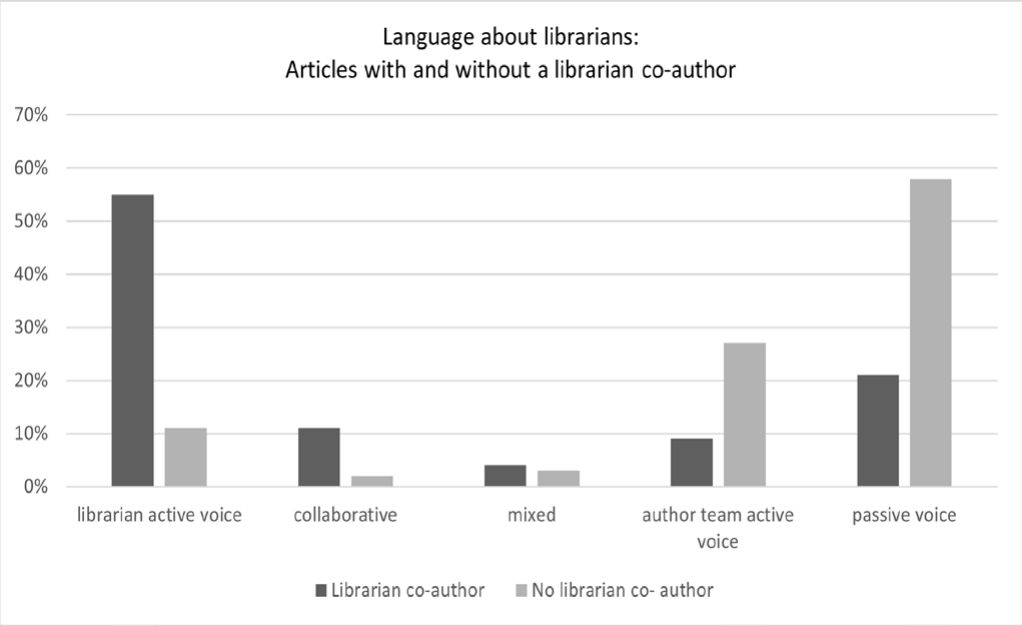
Language about a librarian's role.

When the reviews that described the librarian's work in the body of the review were analyzed by authorship, most of the ones with a librarian co-author (55%, 26/47) described the librarian's work using the active voice, whereas most reviews without a librarian author (58%, 68/117) used the passive voice (see Figure 3). Use of the author team active voice was much more common for reviews without a librarian author (27%, 32/118) than those with a librarian author (9%, 4/47), and use of the collaborative voice was much less common among those without a librarian author (2%, 2/118) than those with a librarian author (11%, 5/47).

### Assessment of search strategy

#### PRISMA chart & number of databases searched

All reviews contained a flow diagram illustrating results numbers, similar to the type described in PRISMA (Preferred Reporting Items for Systematic Reviews and Meta-Analyses), which is a set of guidelines intended to improve the reporting of systematic reviews [21]. However, the exact format and content of some of these diagrams at times varied considerably from the PRISMA template that was available at the time of publication [22].

The number of bibliographic databases searched varied widely, with a small number describing as few as two databases, and some listing nine or more. An average number of databases could not be calculated due to variations in terms of how databases were described, including references to platforms rather than specific databases (e.g., simply saying “EBSCO” or “ProQuest”), and discrepancies in terms of whether a resource was described as a single entity or multiple entities, such as “Cochrane Library” versus CENTRAL and Cochrane Database of Systematic Reviews.

#### Reproducibility of search strategy & use of both keywords and subject headings

**Figure 4:**
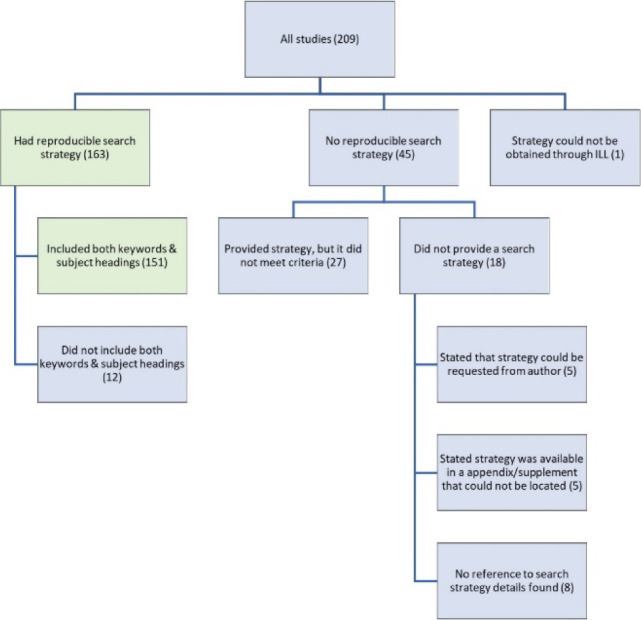
Breakdown of reviews by search strategy.

One-hundred sixty-three (78%) of the articles included a search strategy for a named database using Boolean operators, while 45 did not. One search strategy, which was included in a supplementary file, could not be obtained via interlibrary loan (see Figure 4 for a full breakdown for all reviews).

Of the 45 that did not include a reproducible strategy, 27 provided a search strategy without sufficient information for analysis, and 18 did not provide a search strategy.

Among the 27 reviews that provided a search strategy that was not reproducible, the most frequent issues were no database being specified, and a lack of sufficient information about how terms should be combined.

Of the 163 reviews with a reproducible search strategy, 151 included both keywords and subject headings, and 12 did not. If even a single subject heading was included, then the review was given credit for having both keywords and subject headings.

These two basic aspects of search strategy, reproducibility and use of both keywords and subject headings, were also analyzed by how they corresponded with different types of documentation of the librarian's involvement in the published review. Table 4 shows the breakdown of reproducibility of search strategy and use of keywords and subject headings in reviews with different types of librarian documentation.

**Table 4:** Librarian documentation and search strategies.

Librarian documentation*	Reproducible search strategy	Use of both keywords & subject headings
Librarian coauthor (n=58)	84% (49)	81% (47)
Librarian mentioned in body of the review (n=164)	77% (126)	71% (117)
Librarian in acknowledgments (n= 86)	77% (66)	70% (60)
Librarian not specified by name (n=65)	74% (48)	68% (44)
Librarian not mentioned at all (n=19)	79% (15)	74% (14)

* Total is more than the number of reviews, as some reviews fit into multiple categories

## DISCUSSION

While previous studies have often focused on reviews in a particular health science discipline, in a set of journals, or produced by authors affiliated with a specific institution, and sometimes included less current reviews, the reviews analyzed here came from a wide variety of journals, disciplines, and institutions and were all published since 2017. The librarian authors within the set were also affiliated with an array of international institutions, rather than restricted by specific institution or geographical location.

Our overall finding was that librarians' work in published reviews was typically described in limited terms in relationship to both scope and contribution. Librarians' contributions were usually only described in terms of search strategy development or generic search language. Reviews that did not have a librarian co-author usually described librarian's contributions with language that placed them in the role of assisting/helping with a task rather than actually implementing it, unlike the reviews with a librarian co-author which used more active language. While academic writing often uses passive language rather than naming who did the specific work, this discrepancy may be a revealing one. The use of passive language, intentionally or not, can serve to obfuscate the fact that significant work may have been contributed by someone outside of the authors of the review.

However, some of the language used to describe librarians themselves reflected an awareness among authors that librarian involvement adds to methodological strength. By using words such as “experienced,” “specialist,” and “expertise,” or specifically highlighting the inclusion of a librarian as a strength of the review, authors signaled their awareness of this strength. Recent articles in various disciplinary journals [6,9,23-25] have explained the importance of consulting or collaborating with a librarian when conducting a systematic review, and our study did seem to indicate an awareness of this value in some cases.

The International Committee of Medical Journal Editors (ICMJE) recommends that authorship for a systematic review be based on four criteria [26]. Yet, even when librarians' contributions to a review fulfill all four criteria, this may still not lead to authorship or even acknowledgment [3]. We anticipated that this study, however, would find a higher level of librarian co-authors and acknowledgments/documentation than most previous studies. This was both due to its focus on works that specified librarian involvement at the protocol level, and our initial expectation that publications with authors who had actively updated their PROSPERO record to reflect publication—which many authors fail to do [27-29]—might result in a collection of reviews with authors particularly disposed to adhere to best practices and documentation. Certainly, there was a much higher level of librarian co-authors than in several previous studies. Previous studies, which did not limit to reviews for which there had been prior indication of librarian involvement, found librarian co-authorship ranged from 1-7% of the reviews. [4,6,8]. Ultimately, the rate of librarian co-authors and librarians listed in the acknowledgements were more similar to that found by Koffel's study, which limited its scope to studies where the surveyed authors reported that a librarian was involved in a review [9], and to Ross-White's 2020 update about reviews published by authors associated with Queen's University, whose library has a tiered systematic review support service [12].

Unfortunately, almost one-third of the reviews did not provide the name of the librarian who contributed to the work, and 9% of the reviews did not reference a librarian at all despite having indicated librarian involvement in their PROSPERO protocol. It is certainly plausible that in some of the cases where a librarian was referenced in the protocol but not mentioned in the review, the librarian was not in fact ultimately involved. Unfortunately, it is also plausible that a librarian was involved and their contributions were not documented. While it was not always known in previous studies whether a lack of documentation represented an actual absence of librarian participation, in one study the authors had personal knowledge of librarian involvement that went undocumented [6]. In another study, some systematic review authors themselves stated that a librarian not referenced in the final publication had made a sizable contribution to the work [9].

Search as a form of invisible labor has been explored by Amanda Ross-White, who discussed how librarians' work on systematic reviews can be devalued both by other researchers and by librarians themselves [7]. Named recognition can be used by librarians to show their value to their institution, and their supervisors may assume that a lack of named recognition reflects a lack of involvement with the review, whereas authorship credit may be assumed to reflect a high level of involvement [3]. As such, while negotiating for authorship can be an uncomfortable task for librarians [3] and not all research teams may not be open to such requests [10], librarians should feel justified and supported in advocating for authorship when appropriate. Also, researchers should ideally assuage any potential concerns by actively discussing authorship with their librarian collaborators and ensuring that the final review accurately reflects the librarian's contribution.

Interestingly, all 209 reviews included PRISMA-style flow diagrams, which indicates that this is rightly considered an essential feature of a systematic review. Conversely, a reproducible search strategy does not yet seem to have achieved this same status since this was in only 78% of the publications, and 9% did not include a search strategy at all. As explained by Rethlefsen et al. [6], previously there was not an agreed-upon definition of what constituted a reproducible search. The recent publication of PRISMA-S [30, 31] provides helpful explanations of all components necessary for achieving search reproducibility. While PRISMA-S was not available at the time of publication for the reviews we examined, it is imperative that future review authors rely on this guidance to ensure search reproducibility to the greatest extent possible.

While reviews with a librarian co-author had somewhat higher inclusion of reproducible search strategies and use of both subject headings and keywords, overall this did not vary enormously based on how the librarian's involvement was or was not documented. It was, in fact, concerning how even reviews co-authored by librarians did not always include reproducible search strategies or both keywords and subject headings. Although the original intent was to do only a very basic assessment of search strategy, we unfortunately did notice that a number did not use subject headings and keywords consistently or appropriately, made incorrect use of Boolean operators, and sometimes used parentheses incorrectly or omitted them when they were needed. We also encountered examples of reviews with one search strategy for a database which included subject headings, but then searches for additional databases without subject headings.

These shortcomings raised the question of whether librarians involved in systematic reviews may not always have sufficient training for this mode of research. Similarly, journal editors and peer reviewers may be unaware of best practices for reporting systematic review searches and miss opportunities to improve upon a reported search strategy, or even require that authors present their search strategy in a way that runs counter to recommended practice. Another final possibility is that when librarians are not authors on the final published review, their search strategies may be described secondhand and may contain inaccuracies that the author team does not detect. All these possibilities highlight the value of rigorous peer review of systematic review search strategies by information specialists, as was advocated for in a 2021 letter from six key administrators of international health information associations [32].

It is also worth noting that we encountered challenges in accessing appendixes and supplements. Sometimes these issues were due to them not being included with the review, sometimes because the links took us to a different document, and sometimes because while we were able to easily obtain the full text of the review via interlibrary loan, we ran into issues with obtaining the supplements through interlibrary loan. Potential solutions to this issue could include librarians posting the search strategy in their institutional repository and citing that file, as suggested by Roth and Dean [33], or double checking that the search strategy is properly available when the review is published online. It could also be useful for at least some basic information about the strategy to be provided in the main body of the review. Areas for further research could include a larger data set that would allow for statistical analysis of the strength of potential associations between librarian documentation and such factors as publication year, reproducible search strategies, and use of both subject headings and keywords, as well as factors such as geography or type of institution of the principal investigator that were not explored here.

## CONCLUSION

Even among published reviews where authors had specified librarian involvement within their protocol, these librarian collaborators often “disappeared” from the final publications. Librarians' work was frequently described in passive voice, portrayed as a minimal contribution, was sometimes not mentioned at all, and typically did not result in co-authorship. Ideally, librarians whose contributions do not rise to the level of authorship would still have their work described in full with active language and would be listed in the acknowledgements. Librarians whose contributions are potentially sufficient for authorship would be proactively consulted about whether co-authorship was anticipated or would be appreciated. Overall, given the presumption of librarian involvement, it was also concerning that search strategies were not always reproducible, or even included, and did not always include the use of both keywords and subject headings. It appears that there is much room for improvement in terms of how librarians' involvement is documented, and the level of rigor that systematic review searches adhere to in terms of reproducibility and comprehensiveness.

## Data Availability

Data associated with this article are available in the Open Science Framework at https://osf.io/tgae7/?view_only=769c02ab999e471db69979daf56b1df0.

## SUPPLEMENTAL FILES

**NA**
